# Medical News and Miscellany

**Published:** 1898-02

**Authors:** 


### Medical News and Miscellany.

**Dr. J. M. Witt** has removed from Salado to Bartlett, Texas.

**Dr. P. C. Woods**, of San Marcos, died on January 27, 1898.

**Dr. Sam Cunningham** has removed from Taylor to Dallas.

**Dr. J. D. Gray** has removed from Whitney to Moulton, Texas.

**Dr. A. F. Tucker,** formerly of Galveston, has located at San Angelo, Texas.

---

**Work** for a big meeting of our State Medical Association at Houston in April next. Important matters in medical politics are to be attended to.

---

**Dr. Edward B. Jackson,** of Houston, Texas, desires a young physician partner. Must be sober and energetic. Write or call for particulars.

---

**Dr. H. B. Granberry,** of this city, was married on the 18th of last month (January) to Miss Seale, of Bryan, a sister-in-law of Mr. Morley, the wholesale druggist of Austin.

---

**Dr. S. W. Rimmer,** recently of Richland Springs, Texas, has located in Austin. The JOURNAL extends a welcome to the doctor. He is of the right kind, and Austin welcomes desirable citizens.

---

**Dr. W. E. Pugh,** State quarantine officer at Aransas Pass station, is attending a special course of lectures at Louisville, Ky. He requests the Red Back sent there during his stay. "Can't do without news from home."

---

**The Texas Medical Journal** has on hand a large number of the latest medical works—sent us for review—which can be bought for cash at a discount. Send for list of books and prices. We have nearly all the late medical works.

---

**Dr. Theophilus Parvin,** the eminent obstetrician and gynecologist, of Philadelphia, died at his home last Saturday, aged 72 years. Dr. Parvin was graduated from the University of Pennsylvania in 1852, and at the time of his death he was professor of obstetrics and gynecology at Jefferson Medical College.

**Quinine in Suppositories.**—Quinine, ten to twenty grains, in suppositories per rectum, gives good results without the production of the nausea, headache, singing in the ears, etc. It should be given in this manner in all cases of fever in which quinine is indicated, and especially in malaria and in case of children.—*Dr. T. Dunbar Brunton, in British Medical Journal.*

---

**Every Lover of Science,** every lover of his profession, who reads this should write at once to his representative in the United States Senate protesting against the passage of the infamous bill steered by Senator Gallagher, of New Hampshire. It is a bill aimed at the hated "regulars" by our friends—the homeopaths. Gallagher is a homeopathic doctor. It is known as the antivivisection bill, and is ostensibly to "prevent cruelty to animals." It is, really, to prevent experimental research on animal physiology.

---

**Dr. West's letter** will be read with interest. It is not only a clear vindication of himself against the charge of stultification—for he makes an argument that cannot be answered as to yellow fever not letting go when it seizes on a community; but it is one of the best clinical descriptions of yellow fever yet written. We cheerfully acquit him. We knew that Dr. West was an experienced yellow fever physician, but could not understand how even he could make a *post facto* diagnosis. We are enlightened; and thank the doctor for his letter.

---

**The Attention** of medical societies is called to the suggestion elsewhere published in this issue that the legislature be applied to for a law to set aside at least ten dollars of the proceeds of the crop of every farmer for his medical bill. The landlord and the supply men get the entire crop anyhow, and nothing is left with which to pay the doctor. It would be a just law, and, as our correspondent says, would be of much benefit to the country doctor. We think the suggestion is worth acting on. At the next meeting of the medical society discuss the subject, and start the ball in motion looking to such a measure.

---

**"St. Louis Laryngological and Otological Society."**—On December 27, the St. Louis Laryngological and Otological Society was formed, composed of those physicians of St. Louis who limit their practice to the treatment of diseases of the nose, throat and ear. Dr. J. C. Mulhall was elected president; Dr. J. B. Shapleigh vice-president; Dr. F. M. Rumbold secretary, and Dr. A. S. Barnes, Jr., treasurer, for the year 1898. Meetings will be held monthly, and it is expected that the scientific programs furnished will be highly interesting and instructive. While the membership is limited, the privilege of inviting professional friends is reserved to each member.

---

**The great autocrat** of the Marine Hospital Service—Dr. Wyman—having in violation of the clear letter of the law removed the United States Quarantine back to Ship island, from Chandeleur Island, where it had been put by act of Congress in accordance with the wishes of the Mississippi people,—and by so doing inflicted upon the South the late epidemic of yellow fever—has met with a silent reprimand by Congress. Senator Walthall, of Mississippi, introduced a bill into Congress removing the quarantine station a second time to Chandeleur Islands. Wonder if his highness will gainsay it?

---

**A Good One** on our neighbor of the *Texas Medical News*. In giving an account of the meeting of the Prison Association, and speaking of Dr. Daniel's paper which was read on that occasion, the *News* said: "Dr. Daniel recommended that the United States government in order to prevent overflows of the Mississippi river, construct permanent crevasses instead of temporary ones to be washed away annually." The idea of *washing away a crevasse* is about the most absurd thing conceivable. Get your dictionary, doctor. The suggestion was, to act on the example set by nature; a crevasse or *break in the levee* relieves the pressure and prevents overflow at other points. Make permanent outlets to the flood water and conduct it by canals to adjacent streams, instead of letting it spread out, as when crevasses occur.

